# The importance of active learning and practice on the students' mastery of pharmacokinetic calculations for the intermittent intravenous infusion dosing of antibiotics

**DOI:** 10.1186/1472-6920-12-116

**Published:** 2012-11-22

**Authors:** Reza Mehvar

**Affiliations:** 1 Department of Pharmaceutical Sciences, School of Pharmacy, Texas Tech University Health Sciences Center, 1300 S. Coulter, Amarillo, TX, 79106, USA

**Keywords:** Active learning, Practice opportunities, Evaluation of performance, Pharmacokinetics, Elimination rate constant, Half life, Volume of distribution

## Abstract

**Background:**

Estimation of pharmacokinetic parameters after intermittent intravenous infusion (III) of antibiotics, such as aminoglycosides or vancomycin, has traditionally been a difficult subject for students in clinical pharmacology or pharmacokinetic courses. Additionally, samples taken at different intervals during repeated dose therapy require manipulation of sampling times before accurate calculation of the patient-specific pharmacokinetic parameters. The main goal of this study was to evaluate the effectiveness of active learning tools and practice opportunities on the ability of students to estimate pharmacokinetic parameters from the plasma samples obtained at different intervals following intermittent intravenous infusion.

**Methods:**

An extensive reading note, with examples, and a problem case, based on a patient’s chart data, were created and made available to students before the class session. Students were required to work through the case before attending the class. The class session was devoted to the discussion of the case requiring active participation of the students using a random participation program. After the class, students were given additional opportunities to practice the calculations, using online modules developed by the instructor, before submitting an online assignment.

**Results:**

The performance of students significantly (*P* < 0.001) improved from a baseline of 11.3% (pretest) to 60.3% (posttest) after the class discussion. The grades of students further improved (*P* < 0.001) to 89.3% on the take-home assignment after they had a chance to study on their own and work on the online practices. Finally, students scored 82.6% in a formal mid-term examination, suggesting significant retention of the materials.

**Conclusions:**

Despite being a difficult subject, students achieve mastery of pharmacokinetic calculations for the topic of intermittent intravenous infusion when appropriate active learning strategies and practice opportunities are employed.

## Background

Intermittent intravenous infusion (III) is a mode of drug administration whereby the drug is administered through short intravenous infusions at regular intervals. This method of drug administration may be useful for avoiding dangerously high concentrations that may be achieved by intravenous bolus administration. Additionally, the short infusion time masks the distribution phase, therefore minimizing problems associated with the interpretations of the plasma concentration-time data for drugs that follow multicompartment kinetics.

Important drugs that are administered by the III method are aminoglycosides and vancomycin, which are usually administered by 30–60 min short infusions [1,2]. The initial dose and dosing interval of these drugs are normally determined based on population pharmacokinetic parameters adjusted for the patient’s characteristics, such as creatinine clearance and weight. After the administration of the initial dose, however, drug concentrations are determined in serum samples taken from the patient, and the dosage regimen is adjusted, if necessary. Usually, two samples (peak and trough) are taken after the first dose or at steady state for determination of drug concentrations. For aminoglycosides, the common sampling times for the peak are 30 min after a 30-min infusion or 15 min after a 60-min infusion. Regardless of the length of infusion, it is recommended that the peak sample be taken ≥ 1 h after the start of infusion to avoid the distribution phase [3]. The second (trough) sample is normally taken ≤ 30 min before the next dose is administered. In some cases, the trough is taken immediately before the next infusion is administered. The serum drug concentrations are then used for the estimation of patient-specific kinetic parameters.

**Figure 1:**
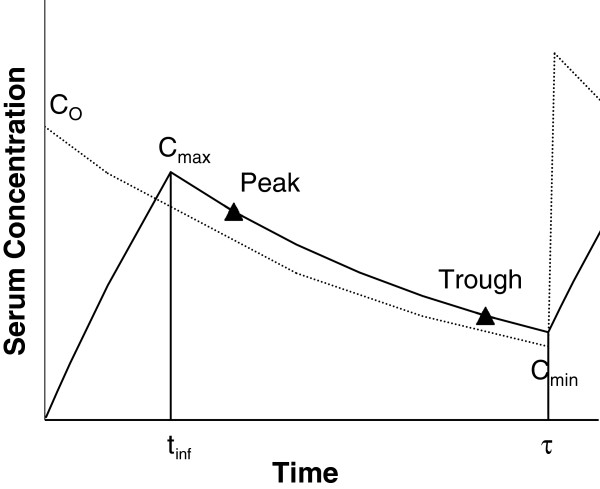
**Plasma**/**serum concentration**-**time profiles of a drug administered by intermittent intravenous infusion or intravenous bolus method.** Solid and dashed lines represent the intermittent intravenous infusion and intravenous bolus methods, respectively. Abbreviations: C_o_ = maximum concentration after the bolus dose; C_max_ = maximum concentration after the short infusion; t_inf_ = length of short infusion; C_min_ = minimum concentration.

There are subtle differences between the III and multiple bolus dosing of drugs in terms of estimation of some pharmacokinetic parameters such as maximum plasma concentration (C_max_) and volume of distribution (V). The main reason for these differences is a relatively significant elimination of the drug during the drug input (short infusion) for the III mode, as opposed to a negligible elimination of the drug during the bolus input. Consequently, the C_max_ after III, which occurs at the end of the short infusion, is always less than the maximum concentration after the intravenous bolus dosing (C_o_), which occurs immediately after the bolus dose (Figure 1). Therefore, the estimation of C_max_ and calculation of volume of distribution after III are more complicated than those after the intravenous bolus dosing.

Additionally, a common practice for sampling at steady state for drugs administered through III is to obtain the peak and trough samples from two subsequent dosage intervals: a trough sample is taken first, followed by the infusion of the next dose and collection of the peak sample [3]. This method is more convenient and expeditious relative to obtaining a peak and a trough sample from the same interval, which requires longer time for collection of both samples. The collection of the peak and trough samples from two different dosage intervals, however, requires manipulation of sampling times before accurate calculation of the patient-specific kinetic parameters. At Texas Tech School of Pharmacy, the principles of these calculations have traditionally been discussed in different didactic courses taught to Doctor of Pharmacy (Pharm.D.) students. However, opportunities to practice these principles before their application during the Advanced Pharmacy Practice Experience rotations have been limited. Therefore, new learning tools were created for inclusion into a basic pharmacokinetics course to allow students to learn how to estimate the patient-specific kinetic parameters from the simulated patient chart data, with a particular emphasis on the use of the peak and trough samples from two different dosage intervals at steady state. The purpose of this article is to present these tools and the assessment of their effectiveness.

## Methods

### Educational context

Basic Pharmacokinetics is a 3-semester-credit-hour course that is offered synchronously, using videoconferencing technology, to the local (Amarillo) and distant (Abilene) campuses twice a week with 75 min of instruction time for each session. Although most of the instructions initiate from the Amarillo campus, where the author resides, a second instructor is located in Abilene to assist the students inside and outside the classroom. The course is offered during the second year (P2) of a 4-year Pharm.D. program and runs throughout the fall semester with 16 weeks of instruction, excluding holidays. The prerequisites for the course are P2 academic standing and successful completion of a Principles of Drug Action course that is offered during the spring semester of the first year. The teaching format of the course is based on the active learning principles applicable to large classrooms, as described in more detail in the following sections. For the fall of 2011, the class had a total enrolment of 152 students, with 114 students on the Amarillo campus and 38 students on the Abilene campus.

The overall outcomes of the course are presented in Table 1. Outcome 3 (Table 1) deals with the estimation of patient-specific pharmacokinetic parameters after any route of administration using a limited number of samples. To achieve this outcome for the III mode of drug administration, a 75-min session and various instructional tools were designed and devoted to this topic. The major outcome and learning objectives for this session are presented in Table 2. The session is scheduled around the middle of the semester after coverage of the pharmacokinetics of single oral and intravenous doses, constant intravenous infusion, and multiple bolus intravenous or oral doses. Therefore, students already have ample opportunity to practice estimation of the rate constant and half life using the data from the same interval after the first dose or at steady state. However, the concept of estimation of the elimination rate constant from a peak and trough belonging to two separate intervals is introduced for the first time during the III session. Other new concepts introduced during this session are the estimations of C_max_, which occurs at the end of short infusion (Figure 1), the time difference between the C_max_ and C_min_, which is less than one dosage interval, and the estimation of V using newly-introduced equations based on the Sawchuk-Zaske method [4]. Additionally, simulated chart data for the times of dosing and sampling are used for the first time in this session.

**Table 1:** Major outcomes for the pharmacokinetics course

1.	Evaluate the primary and secondary drug information literature with regard to the pharmacokinetics and pharmacodynamics of drugs.
2.	Evaluate the basic pharmacokinetics and pharmacodynamic properties of a drug and relate them to the manner in which the drug is used therapeutically.
3.	Estimate patient-specific kinetic parameters for any drug and route of administration from a limited number of biological samples.
4.	Design dosage regimens based on the patient-specific or population (average) pharmacokinetic/ pharmacodynamic data.
5.	Predict the effects of route and/or method of drug administration on the plasma concentration-time profiles using the individual or population (average) kinetic data and judge the appropriateness of dosage form and route of administration.
6.	Predict the effects of changes in the physiological parameters (due to drug interactions, disease states, or special populations) on the pharmacokinetics and plasma concentration-time profile of drugs, and recommend a dosage regimen based on the altered parameters.

**Table 2:** Major outcome and learning objectives for the intermittent intravenous infusion session

1.	Expected outcome:
	1.1 Estimate major kinetic parameters (elimination rate constant or half life, volume of distribution, and clearance) from two or more plasma concentration-time data collected after intermittent intravenous infusion of drugs during the first dose or at steady state.
2.	Learning objectives:
	2.1 Define the applications of intermittent intravenous infusion method.
	2.2 Recognize the differences between the maximum and peak and between the minimum and trough concentrations.
	2.3 Estimate the elimination rate constant and half life from the peak and trough concentrations after the first dose.
	2.4 Estimate elimination rate constant and half life from a trough concentration collected at the end of one dosage interval and a peak concentration collected subsequently after the infusion of the next dose at steady state.
	2.5 Estimate the maximum and minimum concentrations from the peak or trough concentrations and elimination rate constant for the first dose or at steady state.
	2.6 Estimate volume of distribution after the first dose or at steady state.
	2.7 Estimate clearance after the first dose or at steady state.

To achieve the stated outcome and learning objectives (Table 2), the following learning tools were used for this session: reading notes, a practice problem for use during the class session, and an online, take-home assignment with multiple opportunities to practice. These tools are described in the following sections.

### Descriptions of the major elements of the reading note^a^

A reading handout was prepared by the instructor, which was made available to students online via the course website. The reading note starts with the expected outcomes and learning objectives for the lesson (Table 2), followed by the potential applications of the III method (i.e., avoidance of high concentrations and masking of the distribution phase). Next, the potential differences between the peak and C_max_ and between the trough and C_min_ are explained (Figure 1), and the students are cautioned not to use these terms interchangeably. The remainder of the note is devoted to the estimation of the kinetic parameters based on the Sawchuk-Zaske method [4], with specific examples.

#### Estimation of the elimination rate constant (k) and half life (t_1/2_)

First, the simple situation when the peak and trough samples are taken from the same interval is discussed. For this method, the calculation of *k* is based on the following equation:

(1) $$k=\frac{1n\left(\frac{C_{1}}{C_{2}}\right)}{T_{2}-T_{1}}=\frac{1n\left(\frac{C_{\text{peak}}}{C_{\text{trough}}}\right)}{T_{\text{trough}}-T_{\text{peak}}}$$

where T_trough_ and T_peak_ are the sampling time of peak and trough, respectively. Subsequently, t_1/2_ may be obtained using *k*:

(2) $$t_{1/2}=\frac{0.693}{k}$$

**Figure 2:**
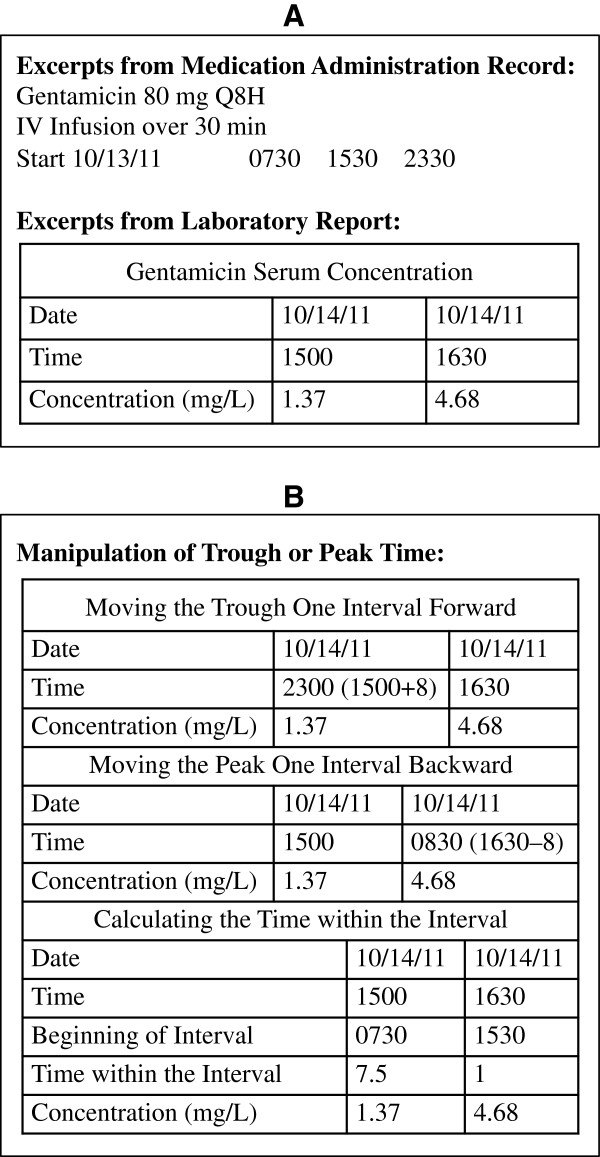
**Chart data.** Excerpts from a patient’s chart data containing the medication administration record and laboratory (plasma concentration-time) data after administration of multiple doses of gentamicin to a patient (**A**) and manipulation of sampling time of trough or peak or calculation of sampling time within the interval for use in equation (1) (**B**).

**Figure 3:**
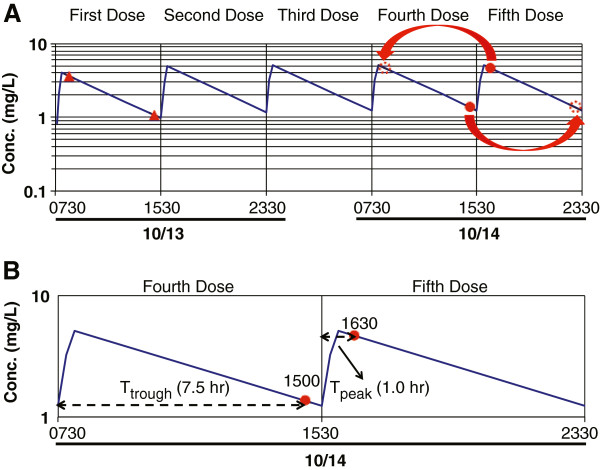
**Serum**/**plasma concentration**-**time Profiles.** Serum/plasma concentration-time course of gentamicin after intermittent intravenous infusion in a patient after intravenous infusion of 80 mg of the drug over 30 min every 8 h, demonstrating the manipulation of peak or trough time (**A**) and calculation of sampling time for peak (T_peak_) and trough (T_trough_) within the interval (**B**). Solid triangles: peak and trough after the first dose; solid circles: peak and trough at steady state; dotted open circles: transposed peak or trough at steady state.

Additionally, a major emphasis is made on the estimation of *k* and t_1/2_ for a case when the trough and peak samples are taken from two different dosage intervals at steady state. In these cases, a trough is first taken around the end of an interval, followed by the administration of the next dose and collection of the peak sample. This is usually done for the sake of convenience and/or expediency. Otherwise, one may have to wait close to one dosage interval to collect both the peak and trough samples from the same dosage interval. An example of data (Figures 2A) obtained from two dosage intervals at steady state (excerpts from the patient’s chart) is presented in the notes. It is obvious that a direct use of the time data given for the peak and trough samples (Figure 2A) in equation (1) would result in an incorrect estimation of *k*. Several solutions for manipulation of time data are then presented to students as demonstrated in Figure 2B. One method is to transform the time of trough or peak. At steady state, the peak and trough concentrations are supposed to be the same for all the dosage intervals, as demonstrated in Figure 3 for an example drug. As shown in Figure 3A, the trough concentration (1.37 mg/L) taken during the 4^th^ interval is the same as that if it had been taken at the same time within the 5^th^ interval (Figure 3A). Therefore, one may assume that the trough sample taken after the 4^th^ dose is indeed the trough sample after the 5^th^ dose, the same interval that the peak was taken. Consequently, the T_trough_ (1500) has to be extended by one dosage interval (8 h) before substitution in equation (1) (Figures 2B and 3A).

Similarly, the peak concentration taken 0.5 h after the infusion is stopped (4.68 mg/L) after the 5^th^ dose is the same as that if it had been taken 0.5 h after the completion of the 4^th^ dose (Figure 3A). Therefore, as an alternative to moving the trough time forward, T_peak_ (1630) may be moved back by one dosage interval (8 h) to the interval when the trough was taken (Figures 2B and 3A) before substitution in equation (1).

Another method for the estimation of T_trough_ and T_peak_ for substitution in equation (1) is to use the sampling time relative to the beginning of the dosing interval in which the sample is taken. In the example here (Figures 2B and 3B), the beginning of dosage interval for the sample taken at 1500 is 0730. Therefore, T_trough_ is 7.5 h (15–7.5). Similarly, the beginning of dosage interval for the sample taken at 1630 is 1530, hence resulting in a T_peak_ value of 1.0 h (16.5-15.5) (Figures 2B and 3B). Substitution of these values in equation (1) results in the same *k* as with the other methods, which transformed the clock time of the peak or trough samples.

#### Estimation of C_max_ and C_min_

In addition to the estimation of *k* and t_1/2_, the note also discusses, with examples, the estimation of C_max_ and C_min_ for any dosage interval (e.g., before and at steady state) using the following general equation:

(3) $$C_{2}=C_{1}e^{-k\left(t_{2}-t_{1}\right)}$$

This equation can be used for the estimation of drug concentration at any time when another concentration-time data (peak or trough) and *k* are known. One must assure, however, that C_2_ is the lower concentration (at the later time of t_2_) and C_1_ is the higher concentration (at the earlier time of t_1_). If the estimated C_max_ and C_min_ are after the first dose, they may be multiplied by the accumulation factor to predict their corresponding values at steady state.

#### Estimation of volume of distribution

The notes also discuss the estimation of V for the first dose, steady state, and dosage intervals between the first dose and steady state using equations (4), (5), and (6), respectively, which are derived based on the Sawchuck-Zaske method [4]:

(4) $$V=\frac{R_{0}}{k}\cdot \frac{\left(1-e^{-kt_{\text{inf}}}\right)}{C_{\text{max}}}$$

(5) $$V=\frac{R_{0}}{k\cdot C_{\text{max}}^{\infty }}\cdot \frac{\left(1-e^{-kt_{\text{inf}}}\right)}{\left(1-e^{-k\tau }\right)}$$

(6) $$V=\frac{R_{0}}{k}\cdot \frac{\left(1-e^{kt_{\text{inf}}}\right)}{C_{\text{max}}-C_{\text{predose}}e^{-kt_{\text{inf}}}}$$

where R_o_, t_inf_, τ, C_max_^∞^, and C_predose_ are the rate of infusion, length of infusion, C_max_ at steady state, and predose C_min_, respectively. Additionally, the degree of error associated with the estimation of V using the bolus dose equations (7) (for the first does) and (8) (for the steady state), listed below, are discussed in the notes with numerical examples [5].

(7) $$V\approx \frac{\text{Dose}}{C_{\text{max}}}$$

(8) $$V\approx \frac{\text{Dose}}{C_{\text{max}}^{\infty }-C_{\text{min}}^{\infty }}$$

The notes state that the degree of overestimation of V using the equations for the bolus route is dependent on the magnitude of difference between C_o_ (if the drug were administered by IV bolus route) and C_max_. This means the longer the length of the infusion or the faster the decline in the plasma concentration (shorter half life), the larger is the difference between the C_o_ and C_max_ values, hence resulting in a higher overestimation of V [5].

#### Estimation of clearance (Cl)

The discussion of the estimation of Cl for this topic in the notes is limited because this calculation (multiplying k by V) is not unique to this route of administration.

### Practice problem for in-class discussion^b^

A practice problem was designed to cover the learning objectives of the lesson (Table 2). The practice problem consisted of two sections, one dealing with the data for the first dose and the other with the steady-state for an antibiotic. For the first dose data, the peak and trough samples obtained after the first dose were presented. For the steady-state data, a trough and peak from two subsequent doses were presented. In both cases, the dose, dosage interval, and start date and times of dosing were described. The students were then asked to estimate the kinetic parameters listed in Table 3 using the presented data. Additionally, they were asked to compare the kinetic parameters (*k*, V, and Cl) obtained after the first dose and at steady state, which are supposed to be similar.

**Table 3:** Questions in the intermittent intravenous infusion practice problem

**Questions for the first dose data**	**Questions for the steady**-**state data**
1. Elimination rate constant and half life	1. Elimination rate constant and half life
2. C_max_ for the first dose	2. C_max_ at steady state
3. C_min_ for the first dose	3. C_min_ at steady state
4. C_max_ at steady state	4. Volume of distribution
5. C_min_ at steady state	5. Clearance
6. Volume of distribution	
7. Clearance	

As with any other topic in the course, students were expected to work on the practice problem, consulting the reading note, before attending the class session.

### Class session

During the first 10–15 min of the class session, the instructor briefly outlined the general concepts of the intermittent intravenous infusion and its potential applications. The remaining time of the session was then devoted to the discussion of the practice problem. The discussion was conducted by calling on students randomly, using an online registration process described previously [6], to answer each question in the practice problem.

### Online homework assignment

An online, interactive module was designed with a structure identical to the in-class practice problem using a system described before [7]. Briefly, the online module would create assignments by randomly selecting the kinetic parameters and the dosing regimen data from a range that is preset by the instructor. Therefore, each student would have his/her own individualized assignment with unique data. The program also allows students to generate unlimited online practices, each with a unique set of data, accompanied by the answers to the questions so that they can practice before submitting their answers to the assignment questions. The students then enter their answers to each question online and receive immediate feedback in the form of the correct answer and grade for that question before they enter the next answer. The assignment was due by the midnight of the day the class session was conducted.

### Assessment

To assess the effectiveness of the tools used to achieve the learning objectives of the session, an online pretest was given to students at the end of the class for the multiple dosing session, which was two days before the session on the III topic. The students did not have access to the notes, practice problem, or the online assignment/practice modules before the pretest. The pretest presented dosage regimen and peak and trough data for the steady state using the simulated chart data for a case when the trough sample was obtained before the next dose peak sample (similar to the data in Figure 2A). The students were then asked to estimate 4 parameters: *k*, C_max_, C_min_, and V. Similar to the online assignments, the pretest for each student had individual, unique data. The students were given 15 minutes to answer the questions. After the pretest, notes, practice problem, and the online assignment/practice modules were made available to students. Two days after the pretest, the III topic was presented to students in a class session, and a posttest was administered at the end of the session using the same questions used in the pretest but with different data set. Again, students were allowed 15 min to complete the test online. In addition to the pre- and posttest, the student grades on the homework assignment for the same questions were compiled. Finally, the grades of the students for the same 4 questions in the mid-term examination, which was administered 3 weeks after the session, was compiled.

A one-way ANOVA with repeated measures (4 assessments), followed by post-hoc Tukey’s multiple comparison, was used to test the differences between the total grades of all students in different assessments. Additionally, to test the effect of location (Abilene and Amarillo) on the performance of students in the 4 assessments, the grades were separated based on the campus. A two-way ANOVA with repeated measures, followed by Bonferroni post-hoc tests, was then used for the statistical analysis of data.

The study was approved by the Institutional Review Board of Texas Tech University Health Sciences Center under the exempt status (IRB#: A11-3674).

## Results

**Figure 4:**
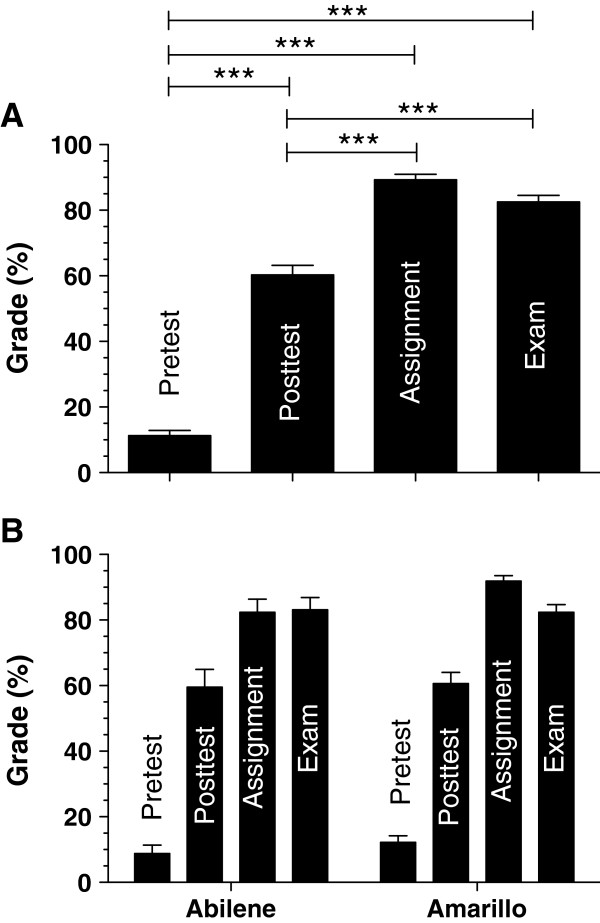
**Performance data for the entire assessments.** Grades of students in Pretest, Posttest, Assignment, and Examination for the entire class (*n* = 126) (**A**) and Abilene (*n* = 34) and Amarillo (*n* = 92) students separately (**B**). Columns and bars represent mean and SEM, respectively. *** *P* < 0.001.

A total of 126 students (34 from Abilene and 92 from Amarillo) completed all 4 assessments, and therefore, were included in the analysis. As demonstrated in Figure 4A, the average grade of students in the pretest was very low (11.3%). However, there was a substantial and significant (*P* < 0.001) increase in the grades at the end of the III session, as demonstrated by an average grade of 60.3% in the posttest (Figure 4A). Compared with the posttest, the average grades of students further increased (*P* < 0.001) to 89.3% when students submitted their take-home assignment. The average grade in the mid-term exam, although slightly, but not statistically, lower than those in the assignment, was significantly (*P* < 0.001) higher than that in the pre- and posttest (Figure 4A). The two-way ANOVA analysis indicated that the performance of the students on the Abilene campus was not significantly different from that of the students on the Amarillo campus (*P* = 0.163) (Figure 4B). Additionally, there were not any significant interactions between the campus location and assessment performance (*P* = 0.443). Therefore, the differences in the grades of students among the 4 assessments for the Abilene and Amarillo students (Figure 4B) were similar to those for the entire class (Figure 4A).

**Figure 5:**
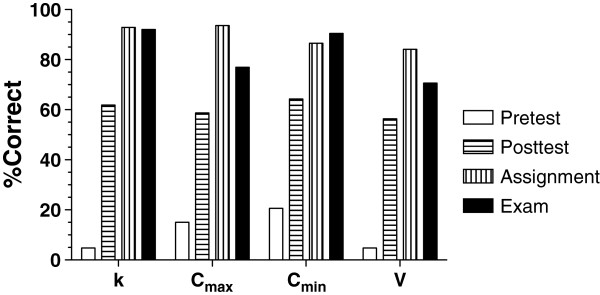
**Performance data for the individual questions.** Percentages of students who answered each of the four questions correctly in Pretest, Posttest, Assignment, and Examination (*n* = 126). Abbreviations: k = elimination rate constant; C_max_ = maximum concentration; C_min_ = minimum concentration; V = volume of distribution.

The percentages of students who answered each of the questions related to *k*, C_max_, C_min_, and V correctly in the 4 assessments are shown in Figure 5. The pairwise comparisons between the assessments were tested using Fisher’s exact test after detection of a significant effect (*P* < 0.0001) of the assessments on the performance using Chi-square analysis. For *k*, the number of students with the correct answer increased progressively (*P* < 0.0001) from the pretest to the posttest and then to the take-home assignment. However, there was no significant difference (*P* =1.000) between the assignment and the exam (Figure 5). Similar observations were also made for the C_min_ question (Figure 5). However, for the C_max_ and V questions, although the performance of the students in the pretest, posttest, and assignments progressively improved, the number of students answering the questions correctly in the mid-term exam was significantly lower than that in the take-home assignment for these two questions (Figure 5).

**Figure 6:**
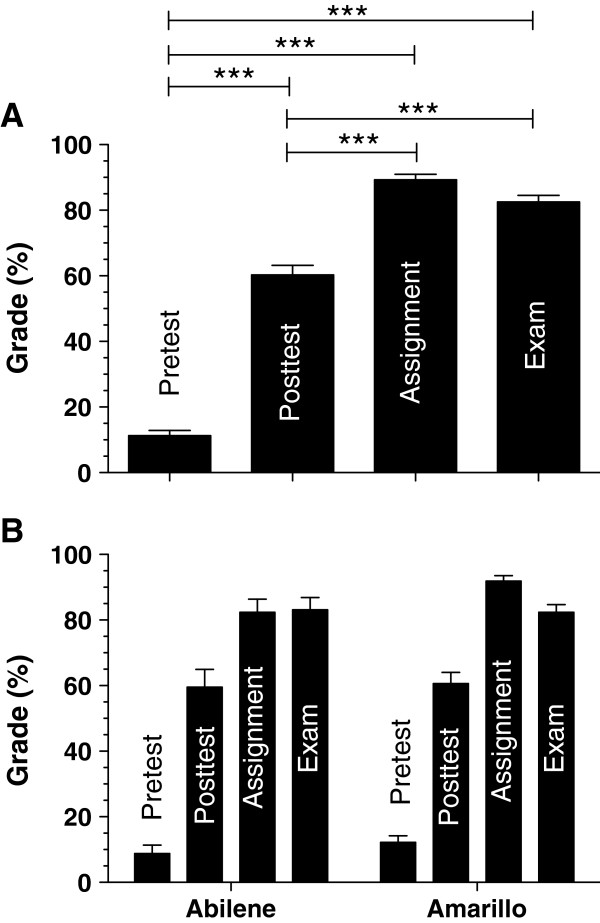
**Effects of online practice generation on the online assignment grade.** Grades of students in the online assignment as a function of the number of online practices generated before the submission of the assignment. The percentages of students who generated zero, one, two, or three practices were 11.9%, 55.5%, 19.0%, and 7.1%, respectively. Symbols and bars represent mean and SEM, respectively.

As for the use of online practices, an overwhelming majority of students (88.1%) generated one or more practices before the submission of the online assignment, as opposed to only 11.9% of the students who did not generate any practices. Of those who generated practices, 55.5%, 19.0%, and 7.1% generated one, two, or three practices, respectively. The remaining students (6.3%) generated ≥ 4 practices. The linear regression analysis of the assignment grades against the number of generated practices for students who generated 0–3 practices (94% of students) are presented in Figure 6. As demonstrated in this figure, there was a significant (*P* < 0.05) positive relationship between the number of generated practices and the assignment grade. The slope of the regression line was 2.5%, indicating that on average every additional practice resulted in a 2.5% increase in the students’ grades within the practice range of 0–3.

## Discussion

The main goal of the instructor for the III session was to design learning tools for students so that they learn how to estimate major patient-specific pharmacokinetic parameters after this route of administration. To accommodate the requirements of some Advanced Pharmacy Practice Experience rotations that students be able to estimate the kinetic parameters using the patient chart data and plasma concentrations taken from two intervals, learning tools were designed with special emphasis on these requirements. The performance data presented in Figures 4 and 5 suggest successful achievement of this goal using the instructional tools developed for this topic. The students’ use of the reading notes and the practice problem on their own before attending the class combined with the activities during the class session resulted in a significant improvement in the performance of the students, which was demonstrated by a substantial improvement (*P* < 0.001) in the posttest grades (60%), compared with the pretest grades (11%). This suggests the effectiveness of the reading notes, practice problem, and classroom activities. Additionally, a significant improvement in the students’ performance in the take-home assignment (89%), compared with the posttest (60%), suggests the effectiveness of the designed take-home assignment with the immediate feedback and unlimited opportunity to practice.

It could be argued that the students’ performance in the take-home assignment is inflated relative to the other tests because of two main differences between the assignment and other tests. First, other than a due date and time, the assignment lacked the time stress present for the other tests. Therefore, students could spend as much time as they needed for each question while answering the assignment questions. Second, whereas the pretest, posttest, and mid-term exam were not open book (except for provision of an equation sheet), students had access to all course materials for the take-home assignment. Therefore, one might expect that the performance of the students in the mid-term exam to be lower than that in the assignment. Although the grades of students in the mid-term exam (83%) were lower than those in the assignment (89%), this difference did not reach statistical significance (Figure 4A). Nevertheless, the significant improvement in the grades between the posttest and mid-term exam (Figure 4A) indicates that the events between the two assessments, including the students’ work on the assignment, improved students’ learning of the subject.

At Texas Tech School of Pharmacy, the courses offered during the first and second year of the pharmacy curriculum are delivered synchronously to Amarillo and Abilene campuses. The instruction may be initiated from either campus depending on the location of the instructor. For the Pharmacokinetics course, all the sessions initiated from Amarillo. Therefore, the Abilene campus was considered the distant campus. To assure equality of performance between the two campuses, all the performance data were routinely monitored separately for the two campuses. As shown in Figure 4B, there were no significant differences between the two campuses in terms of performance in the tests, and the performance data for each site mirrored that for the whole class (Figure 4A).

The performance data on the individual questions (Figure 5) were surprising. Although students had ample opportunity to calculate *k* or half life using the data from the same interval and were familiar with the concept of repeating peak and trough values at steady state, only 4.8% of them were able to calculate *k* correctly in the pretest using the data from two intervals. Despite this very low performance in the pretest, the educational activities designed in this course resulted in an astonishing 93% correct answer in the take-home assignment (Figure 5). Interestingly, this high level of performance was retained even under the time-sensitive and non-open book environment of the mid-term exam, when students scored 92% for answering *k* correctly (Figure 5). This data clearly show the importance of practice in learning pharmacokinetic calculations or concepts. If we, as instructors, would like students know how to estimate a *k* value based on a peak and trough from two separate intervals, we must create opportunities for them to practice this method before assessing them. Nevertheless, the data in Figure 5 clearly show that having utilized the learning tools described here, a large number of students (>92%) are capable of estimating *k* using samples from two intervals under the time-sensitive and stressful conditions of formal examinations.

Although the trend for the C_min_ data was similar to that for *k* in that the performances of students in the take-home assignment and mid-term exam were similar, the number of students who answered the C_max_ or V questions correctly in the mid-term exam was significantly lower than that in the take-home assignment (Figure 5). Indeed, the lowest performance during the mid-term exam belonged to the V question as 70% of students answered this question correctly. This is most likely related to the complexity of the equations for the estimation of V. Therefore, manual calculation of V using the complicated equations (5) or (6) under time-sensitive conditions, such as formal exams, is associated with more error than estimation of other parameters, which use simpler equations.

## Conclusions

In conclusion, learning tools used in a basic pharmacokinetics course offered to second-year pharmacy students are presented here for the topic of intermittent intravenous infusion. In addition to the estimation of pharmacokinetic parameters from the plasma concentrations obtained during one interval, emphasis was placed on a unique case when peak and trough are obtained from two separate dosage intervals at steady state. Additionally, simulated chart data were used to present the dosing schedules and laboratory data. Although students were familiar with the estimation of elimination rate constant before the introduction of the intermittent intravenous infusion topic, >95% of them could not estimate the elimination rate constant accurately when the peak and trough samples were from two separate intervals. Active learning exercises and practice opportunities employed for this topic substantially facilitated students’ mastery of these calculations, as evidenced by performance data.

## Endnotes

^a^A complete copy of the reading note is available from the author by request.

^b^A complete copy of the practice problem is available from the author upon request.

## Competing interests

The author reports no competing interest.

## Authors’ contributions

RM designed and carried the study, analyzed the data, and wrote the manuscript.

## Authors’ information

RM, PharmD, PhD, is a Professor of Pharmaceutical Sciences who has taught basic and clinical pharmacokinetics courses to pharmacy students for over 25 years. He has developed numerous active learning strategies for teaching pharmacokinetics, for which he has received several awards including Innovation in Teaching Award from the American Association of Colleges of Pharmacy and President’s and Chancellor’s Awards in Teaching from the Texas Tech University Health Sciences Center.

## Pre-publication history

The pre-publication history for this paper can be accessed here:

http://www.biomedcentral.com/1472-6920/12/116/prepub
